# Complete Symptom Resolution After Rest in Acute Dizziness: Implications for Selective Magnetic Resonance Imaging Use and Patient Out-of-Pocket Costs in Japan

**DOI:** 10.3390/healthcare14213784

**Published:** 2026-11-05

**Authors:** Kazuki Kikuchi, Keisuke Suzuki, Kaede Hiruma, Yoko Tarumi, Akihito Kato, Naoki Uchida, Kenji Dohi

**Affiliations:** 1 Department of Emergency, Critical Care and Disaster Medicine, Showa Medical University, 1-5-8 Hatanodai, Shinagawa-ku, Tokyo 142-8666, Japan; kikuchikazuki@gmail.com (K.K.); ka07202251@med.showa-u.ac.jp (K.S.); khiruma@med.showa-u.ac.jp (K.H.); akihito@med.showa-u.ac.jp (A.K.); 2 Clinical Research Institute for Clinical Pharmacology and Therapeutics, Showa Medical University Karasuyama Hospital, 6-11-11 Kitakarasuyama, Setagaya-ku, Tokyo 157-8577, Japan; nuchida@med.showa-u.ac.jp; 3 Department of General Medicine, Showa Medical University, 1-5-8 Hatanodai, Shinagawa-ku, Tokyo 142-8666, Japan; tarumi-y@med.showa-u.ac.jp

**Keywords:** dizziness, vertigo, emergency department, magnetic resonance imaging, symptom resolution, medical expenditure, out-of-pocket payments, healthcare resource allocation

## Abstract

**Background/Objectives**: We examined the association between symptom resolution after rest and causative central lesions on initial magnetic resonance imaging (MRI), and the patient-perspective economic implications of hypothetical MRI omission. **Methods**: This exploratory, single-center, retrospective observational study included 170 patients with dizziness who underwent MRI between April 2022 and March 2024. Symptoms were reassessed after 10 min of supine rest. Among patients discharged home, MRI-related charges were hypothetically omitted for those with complete resolution, with other care unchanged. Sensitivity analyses examined the impact of hypothetical additional outpatient revisit costs. **Results**: Causative central lesions were detected in 0 of 90 patients with complete resolution (0.0%; 95% confidence interval [CI], 0.0–4.0%) and 12 of 80 with residual symptoms (15.0%; 95% CI, 8.0–24.7%; *p* < 0.001). Among 89 discharged patients, hypothetical MRI omission in 54 (60.7%) reduced estimated initial medical expenditures by JPY 909,300 (22.6%); the median estimated patient-payment reduction was JPY 3552. In sensitivity analyses limited to additional outpatient revisits, estimated reductions remained positive across the principal scenarios but diminished as assumed revisit use increased. **Conclusions**: Complete symptom resolution was associated with a lower observed proportion of causative central lesions and warrants evaluation as information complementary to established assessment. Hypothetical selective MRI use reduced estimated initial expenditures and patient payments. These exploratory findings do not establish the safety of MRI omission; prospective validation incorporating clinical outcomes and downstream healthcare use is required.

## 1. Introduction

Dizziness accounts for 3.3% of emergency department (ED) visits in a nationally representative US study [1]. Although many causes are benign, stroke or transient ischemic attack was diagnosed in 3.2% of patients aged 45 years or older presenting with dizziness, vertigo, or imbalance [2]. Distinguishing peripheral vestibular disorders from central causes remains challenging. The Guidelines for Reasonable and Appropriate Care in the Emergency Department 3 (GRACE-3) recommend syndrome-based assessment, including the head impulse, nystagmus, and test of skew (HINTS) examination for appropriately selected patients [3]. However, accurate use requires training, and implementation in routine emergency care remains difficult [4,5].

Neuroimaging contributes to the economic burden of dizziness care [6,7]. In Japan, a nationally uniform fee schedule regulates payment for insured services, while copayment arrangements determine the amount borne by patients [8]. Household data indicate that financial hardship remains a concern despite universal coverage [9], and a Japanese study of benign paroxysmal positional vertigo (BPPV) identified imaging expenditure at referring institutions [10]. Broader socioeconomic and environmental correlates of healthcare expenditure have also been reported [11]. Because total medical expenditure does not fully represent patients’ financial burden, separate assessment of out-of-pocket payments can complement economic evaluations [12,13].

Practical information that complements established bedside assessment may help inform imaging decisions [3,14,15]. Reports of symptom duration and triggers are more consistent than descriptions of dizziness quality, supporting the emphasis on timing, triggers, and targeted examinations in the TiTrATE framework [16,17]. Whether complete symptom resolution at a brief reassessment is associated with a lower proportion of causative central lesions on initial magnetic resonance imaging (MRI) remains the question addressed here. In this exploratory, single-center, retrospective observational study, we compared patients with complete symptom resolution and residual symptoms after 10 min of supine rest. As a secondary objective, we estimated initial medical-expenditure and patient-payment changes under hypothetical MRI omission among patients discharged home, and examined how additional outpatient revisit costs could offset these reductions.

## 2. Materials and Methods

### 2.1. Study Design and Participants

This exploratory, single-center, retrospective observational study used medical records and billing data. The study was conducted in the ED of Showa Medical University Hospital, which provides primary and secondary emergency care. We retrieved all records of ED visits for which dizziness was the chief complaint between 1 April 2022, and 31 March 2024. Visits were eligible if a peripheral vestibular or central nervous system disorder was suspected during the initial ED assessment. Visits attributed to other causes, including cardiovascular disease, metabolic or electrolyte disturbances, infection, bleeding or anemia, medication effects or poisoning, trauma, heat illness, psychogenic causes, and cervicogenic dizziness, were excluded. We also excluded visits accompanied by headache, cochlear symptoms, or overt focal neurological deficits at presentation. Additional exclusion criteria were inability to ascertain symptom status after 10 min of rest from the medical records and absence of head MRI. Each patient’s eligible ED visit was included in the analysis, and no patient had more than one eligible visit. No formal sample size calculation was performed. The study size was determined by all patients who met the eligibility criteria and underwent initial head MRI during the predefined study period. The analysis was exploratory and was not designed to establish a prespecified safety threshold for MRI omission. The study is reported in accordance with the Strengthening the Reporting of Observational Studies in Epidemiology (STROBE) statement [18].

### 2.2. Assessment of Symptoms After Rest

After arrival, patients rested in the supine position for 10 min. This was a practical reassessment interval incorporated into routine initial evaluation, not a physiologically validated threshold. Temporal symptom information is emphasized in the TiTrATE approach to acute dizziness [17]. History taking and physical examinations that could be performed without interrupting the rest period were performed during this period, after which patients were asked about their subjective dizziness. Based on the medical records, patients were classified into two groups: complete resolution, defined as complete disappearance of subjective dizziness after rest, and residual symptoms, including improved but unresolved symptoms and persistent symptoms. No standardized symptom severity scale was used; classification relied on the documented patient report. Medication administration and imaging were performed after symptom changes had been assessed.

### 2.3. MRI and Final Diagnosis

Patients with suspected peripheral vestibular or central nervous system disorders at the initial ED assessment generally underwent head MRI within 24 h of arrival. MRI was performed using a 1.5-T scanner (MAGNETOM ESSENZA; Siemens Shenzhen Magnetic Resonance Ltd., Shenzhen, China). The presence or absence of central lesions was determined based on the initial head MRI. Images were interpreted by radiologists who were blinded to the symptom assessment after rest. The physician who established the final diagnosis determined whether an MRI-detected lesion was related to the presenting dizziness based on the clinical presentation and imaging findings. A central lesion was considered present when it was judged to be the cause of dizziness. Findings from repeat MRI were not used to determine the primary outcome.

The final diagnosis was obtained from the clinical department responsible for evaluating the patient’s dizziness. For patients referred by an emergency physician to another department, the diagnosis established by that department was used; otherwise, the emergency physician’s diagnosis was used. The need for consultation was determined by the treating emergency physician as part of routine care.

Admission for observation was generally recommended in accordance with institutional practice. Patients who preferred outpatient follow-up were discharged from the hospital.

For patients classified as having no central lesions on initial head MRI, we assessed whether the first reassessment by a clinical department relevant to the diagnosis was documented, the number of days from ED arrival to that reassessment, and whether a new central nervous system diagnosis was made at the first reassessment. Reassessments included inpatient and outpatient consultations recorded at this institution. Reassessment on the day after ED arrival was recorded as occurring 1 day later. An unconfirmed reassessment was not treated as evidence of an event-free course.

### 2.4. Data Collection

#### Clinical and Cost Data Collection

We collected data on age, sex, vital signs at presentation (systolic blood pressure, pulse rate, body temperature, respiratory rate, and peripheral oxygen saturation [SpO_2_]), symptom status after rest, disposition after the ED visit (admission or discharge home), and final diagnosis (peripheral vestibular or central nervous system disorder and specific diagnosis), together with the department that established the diagnosis.

Patient characteristics, clinical course, and final diagnoses were summarized for the overall study population and separately for the complete-resolution and residual-symptom groups.

For patients discharged home who were included in the cost analysis, we collected billed fee-schedule points, the amount charged to the patient at the point of care, hospital-designated out-of-pocket surcharge for after-hours emergency care (sentei ryoyohi), and the patient’s copayment rate. We also collected head computed tomography (CT) data and records of after-hours surcharges required to estimate MRI-related expenditures.

### 2.5. Data Sources and Abstraction

Information on symptoms, examination findings, imaging findings, diagnoses, and reassessments was obtained from existing medical records. Cost information was obtained from records of billed fee-schedule points and amounts charged to patients at the point of care. One investigator extracted the information for each patient and confirmed that they had not participated in the patient’s diagnosis. Symptom status after rest was classified based on the recorded symptom information without access to the MRI results or final diagnosis. MRI results and final diagnoses were abstracted only after symptom classification had been finalized. This sequential review was intended to reduce potential review bias in retrospective chart abstraction, consistent with methodological guidance for emergency medicine chart review studies [19].

### 2.6. Outcomes and Cost Analysis of Hypothetical MRI Omission

#### 2.6.1. Primary Outcome

The primary outcome was the proportion of patients with a central lesion detected on initial head MRI that was considered responsible for the presenting dizziness. This proportion was compared between the complete-resolution and residual-symptom groups.

#### 2.6.2. Secondary Outcomes

The secondary analysis included patients in the primary analysis cohort who were discharged directly from the ED without admission (hereafter referred to as patients discharged home). All patients in this cohort had actually undergone initial head MRI. We specified a hypothetical scenario in which initial head MRI was omitted in the complete-resolution group but retained in the residual-symptom group. We estimated the number of MRI examinations that would be omitted, and the resulting reductions in initial emergency care expenditures and patient out-of-pocket payments. All other investigations, treatments, and discharge decisions were assumed to remain unchanged. Hypothetical MRI omission was based solely on symptom status after rest, without consideration of MRI findings or the final diagnosis.

The percentage reduction in MRI use was calculated as the number of omitted examinations divided by the total number of initial MRI examinations performed among patients discharged home. The base-case cost analysis was restricted to initial ED visits and excluded inpatient care and care after discharge. A separate sensitivity analysis assessed hypothetical incremental outpatient revisit costs, as described below.

Medical expenditure was defined as the total fee-schedule charges for insured medical services and was calculated from the actual billed fee-schedule points, with 1 point equivalent to JPY 10. Expenditure under the hypothetical MRI omission scenario was estimated by subtracting MRI-related amounts from actual expenditure using the 2022 Japanese medical fee schedule and its billing instructions, which were applicable during the study period [20,21]. Deductions accounted for the use of a 1.5-T scanner and concurrent examinations and were limited to items that would no longer have been billable if MRI had been omitted. Because itemized claims for individual MRI-related services were not reviewed, these were scenario-based estimates using specified billing assumptions. We confirmed that patients included in the cost analysis had not undergone CT or MRI during the same calendar month before the ED visit. Table S4 presents the specific MRI-related deductions, billing assumptions, and number of patients meeting each condition. Baseline patient out-of-pocket payments were defined as the amount charged for insured medical services after excluding sentei ryoyohi from the actual point-of-care charges. Out-of-pocket payments under the MRI omission scenario were estimated by subtracting the MRI-related deduction multiplied by the individual patient’s copayment rate from the baseline amount and rounding to the nearest yen. Estimated payments were constrained to a minimum of JPY 0. Patients with actual out-of-pocket payments of JPY 0 were retained in the analysis. Public subsidies and subsequent reimbursements that could not be established from the available data were not incorporated into the estimates. The analytical scope, initial-visit time horizon, included costs, and scenario assumptions were reported with reference to relevant CHEERS 2022 guidance [22].

#### 2.6.3. Deterministic Sensitivity Analyses of Added Outpatient Revisits

We conducted a two-stage deterministic sensitivity analysis among the 54 discharged patients whose initial MRI would have been omitted. First, we divided the initial modeled reductions by 54 to determine the mean additional cost per candidate that would offset them. Second, we modeled one additional unplanned outpatient revisit at the same institution under same-calendar-month billing conditions, with no intervening CT or MRI and no admission. Each revisit comprised consultation, blood testing, and noncontrast 1.5-T head MRI. Costs reflected individual initial CT status and copayment rates under four visit-time conditions: weekday daytime, after hours, holidays, and late night. The 2022 fee-schedule components, blood-test panel, and monthly billing assumptions are detailed in Table S5.

For each time condition, the remaining reduction equaled the initial reduction minus the assumed additional revisit proportion multiplied by the sum of patient-level revisit costs, calculated separately for medical expenditures and patient payments. The same proportion was applied to all candidates: 0% as the baseline; 1%, 2%, 5%, 10%, and 20% as principal scenarios; and 30–100% as stress tests to identify break-even thresholds. These author-specified scenarios examined cost-offset conditions rather than predicting actual revisits; individual revisit dates were not simulated.

### 2.7. Statistical Analysis

Continuous variables are presented as medians [first quartile–third quartile], and categorical variables as numbers and percentages. Unless otherwise specified, percentages were calculated using the number of patients with evaluable data for the relevant variable as the denominator, with missing counts reported separately. Missing values were not imputed, and each analysis included patients for whom the required information was available.

Patient characteristics and clinical information were summarized descriptively for the overall population and separately for the two symptom groups. No between-group hypothesis tests were performed for baseline characteristics. Diagnoses and clinical departments were summarized as numbers and percentages. Because no patient contributed more than one visit, each patient was treated as an independent observation.

For the primary analysis, the proportion of patients with a central lesion and its two-sided exact 95% confidence interval (CI) were calculated for each symptom group using the Clopper–Pearson method. The proportions were compared using a two-sided Fisher’s exact test, with a significance level of 0.05. No multivariable adjustment was performed.

For the secondary analysis, the hypothetical scenario was applied to patients discharged home to calculate the number and percentage of MRI examinations that would be omitted. Medical expenditures and patient out-of-pocket payments were summarized as totals under actual care and under the hypothetical scenario, together with absolute and percentage reductions. Medians and interquartile ranges were also calculated for per-patient costs among all patients discharged home and for per-patient reductions among patients for whom MRI would be omitted. Quartiles were calculated using the R quantile function with type = 7. Because the cost analysis comprised scenario-based estimates under specified billing assumptions, neither significance tests nor CIs were calculated for cost differences.

For the added-revisit sensitivity analysis, break-even proportions were calculated by dividing the initial modeled reductions by the sum of added patient-level revisit costs in each scenario. Calculations were performed using unrounded patient-level values; displayed monetary values and proportions were rounded for reporting.

Statistical analyses were performed using R version 4.6.1 (R Foundation for Statistical Computing, Vienna, Austria). Exact 95% CIs for proportions were calculated using the binomial test, and between-group comparisons were performed using Fisher’s exact test, both implemented in the stats package.

### 2.8. Ethical Considerations

The study was conducted in accordance with the Declaration of Helsinki and was approved by the Ethics Committee of Showa Medical University on 17 December 2025 (approval no. 2024-159-B). Individual informed consent for participation was not obtained. In accordance with the study protocol approved by the Ethics Committee, information about the study was made publicly available, and patients were given the opportunity to decline participation through an opt-out procedure. Anonymized patient data were analyzed.

### 2.9. Generative Artificial Intelligence Use

Generative artificial intelligence (ChatGPT, GPT-5.6 Sol; OpenAI, San Francisco, CA, USA) was used during manuscript preparation to assist with translation and language refinement, organization and restructuring of manuscript text, and development and verification of statistical analysis code. The tool was not used to generate or modify the original study data or to make clinical diagnoses or outcome classifications. All statistical analyses were performed by the authors using the statistical software specified above, and all AI-assisted outputs, statistical code, analyses, interpretations, and manuscript text were independently reviewed and verified by the authors. All authors are responsible for the accuracy and integrity of the final manuscript.

## 3. Results

### 3.1. Patient Selection and Characteristics

A total of 418 patients with dizziness were identified. After excluding 101 patients whose dizziness was attributed to causes other than peripheral vestibular or central nervous system disorders, 317 patients remained. A further 88 patients were excluded because of headache, cochlear symptoms, or focal neurological deficits, leaving 229 patients. Five patients had undocumented symptom status after rest, and 54 of the remaining 224 patients did not undergo initial head MRI. The final analysis included 170 patients (Figure 1).

The median age was 71 years [57–80], and 51 patients (30.0%) were male. Complete symptom resolution after rest was documented in 90 patients (52.9%), and residual symptoms were documented in 80 patients (47.1%). The median age was 73 years [60–80.8] in the complete-resolution group and 65.5 years [55.8–79.3] in the residual-symptom group; 25 (27.8%) and 26 (32.5%) patients, respectively, were male. Patient characteristics and clinical information are presented in Table 1.

There were no missing data for age, sex, vital signs, symptom classification after rest, or the presence of central lesions on the initial head MRI. Overall, 81 patients (47.6%) were admitted and 89 (52.4%) were discharged. Admission occurred in 36 patients (40.0%) in the complete-resolution group and 45 of 80 patients (56.3%) in the residual-symptom group (Table 1).

Among the 54 patients excluded because MRI was not performed, the recorded reasons were inability to remove a ring in 1 patient (1.9%), a tattoo in 1 (1.9%), implanted metal, such as a pacemaker, in 3 (5.6%), claustrophobia in 3 (5.6%), and patient preference in 46 (85.2%) (Table S2A). The category of patient preference does not establish whether symptom severity, recovery, or other factors influenced the decision not to undergo MRI. Among patients without initial MRI (*n* = 54), complete symptom resolution was documented in 32/54 (59.3%), compared with 90/170 (52.9%) among those who underwent MRI; hospital admission occurred in 11/54 (20.4%) and 81/170 (47.6%), respectively. Median age (69 vs. 71 years) and the proportion of male patients (29.6% vs. 30.0%) were similar in these descriptive comparisons, for which no between-group hypothesis tests were performed (Table S2B).

### 3.2. Central Lesions Detected on Initial Head MRI

Causative central lesions detected on initial head MRI were present in 0 of 90 patients in the complete-resolution group (0.0%; 95% CI, 0.0–4.0%) and in 12 of 80 patients in the residual-symptom group (15.0%; 95% CI, 8.0–24.7%). The proportion was significantly lower in the complete-resolution group than in the residual-symptom group (two-sided Fisher’s exact test, *p* < 0.001; Figure 2).

The 12 patients (7.1%) with central lesions on initial head MRI included 6 with cerebral infarction, 3 with brain tumors, and 1 each with vertebral artery dissection, cerebral hemorrhage, and basilar artery stenosis. Among the 158 patients without a central lesion, the final diagnoses were benign paroxysmal positional vertigo (BPPV) in 106, vestibular neuritis in 17, Ménière disease in 6, and peripheral dizziness of unknown origin in 29 patients (Table S1A). BPPV accounted for 80 of 90 diagnoses (88.9%) in the complete-resolution group and 26 of 80 (32.5%) in the residual-symptom group. The departments that established the final diagnoses and the departments to which patients were admitted are listed in Table S1B.

### 3.3. Reassessment and Medical Record Review

Among the 158 patients classified as having no central lesions on the initial head MRI, the first reassessment by a department relevant to the diagnosis was documented in 125 (79.1%) and could not be confirmed in 33 (20.9%). No new central nervous system diagnoses were identified during the first reassessment of the 125 reassessed patients. The median time to the first reassessment was 1 day [1–1] (range, 1–12 days), and reassessment within 7 days was documented in 124 patients (78.5%). The first reassessment was documented in 72 of 90 patients (80.0%) in the complete-resolution group and 53 of 68 (77.9%) in the residual-symptom group (Table S3).

All 70 admitted patients without central lesions were reassessed the following day. Among the 88 patients discharged without a central lesion, reassessment was documented in 55 (62.5%), with a median time to first reassessment of 1 day [1–3] (range, 1–12 days). Thus, reassessment could not be confirmed in 33 of the 88 patients discharged home (37.5%). Reassessment within 7 days was documented in 54 of 88 patients (61.4%) who were discharged home (Table S3). Among the 54 discharged patients with complete symptom resolution who were candidates for hypothetical MRI omission, a first reassessment was documented in 36 (66.7%) (Table S3).

### 3.4. Hypothetical MRI Omission and Estimated Costs Among Patients Discharged Home

Of the 89 patients discharged, 54 had complete symptom resolution and 35 had residual symptoms (Figure 1). Under the hypothetical scenario in which initial MRI was omitted in the complete-resolution group, the number of MRI examinations would decrease from 89 to 35, representing a reduction of 54 examinations (60.7%) (Table 2).

Cost data were complete for all 89 patients who were discharged. The copayment rates for insured medical services were 0% in 5 patients (5.6%), 10% in 25 (28.1%), 20% in 17 (19.1%), and 30% in 42 (47.2%) (Table 1). Hospital-designated out-of-pocket surcharges for after-hours emergency care (sentei ryoyohi) were charged to 54 patients, totaling JPY 475,200. After these charges were subtracted from the total point-of-care charges of JPY 1,326,262, patient out-of-pocket payments for insured medical services totaled JPY 851,062. The analysis included 5 patients with zero out-of-pocket payments, 2 of whom would have had MRI omitted under the hypothetical scenario.

Total medical expenditures were estimated to decrease from JPY 4,016,030 under actual care to JPY 3,106,730 under the hypothetical scenario, a reduction of JPY 909,300 (22.6%). Total patient out-of-pocket payments were estimated to decrease from JPY 851,062 to JPY 676,630, a reduction of JPY 174,432 (20.5%). Among the 54 patients for whom MRI would be omitted, the median per-patient reductions in medical expenditures and out-of-pocket payments were JPY 11,840 [11,840–23,500] and JPY 3552 [2240–4248], respectively (Table 2).

Across all 89 patients discharged, the median per-patient medical expenditure was JPY 44,660 [38,020–51,050] under actual care and JPY 36,720 [22,850–45,200] under the hypothetical scenario. The median out-of-pocket payments were JPY 8930 [5780–13,640] and JPY 6562 [3290–11,228], respectively (Table 2).

### 3.5. Deterministic Sensitivity Analyses of Added Outpatient Revisits

The mean additional cost per MRI-omission candidate required to offset the initial-visit reduction was JPY 16,839 for medical expenditures and JPY 3230 for patient out-of-pocket payments (unrounded thresholds, JPY 16,838.89 and JPY 3230.22). Assuming a same-calendar-month added outpatient revisit with blood testing and MRI, the mean added medical expenditure per revisit ranged from JPY 18,863 (weekday daytime) to JPY 26,163 (late night), and the mean added out-of-pocket payment ranged from JPY 3622 to JPY 5055 across the four tariff scenarios (Table S5).

Across the principal scenarios assuming additional outpatient revisit proportions of 1–20%, the remaining modeled reductions in medical expenditures and patient out-of-pocket payments were positive under all four time-of-visit conditions (Table 3). The additional revisit proportion required to offset the initial reduction ranged from 89.27% to 64.36% for medical expenditures and from 89.17% to 63.90% for patient payments, depending on the time-of-visit scenario (Table S5). These are conditional modeling results, not observed revisit frequencies or full-episode net savings.

## 4. Discussion

In this study of 170 patients with acute dizziness who underwent MRI, complete symptom resolution after 10 min of rest was associated with a lower proportion of causative central lesions on initial MRI: 0/90 compared with 12/80 among patients with residual symptoms. Among the 89 patients discharged home, hypothetical MRI omission in the 54 patients with complete resolution reduced estimated initial medical expenditures by 22.6% and patient out-of-pocket payments by 20.5%. Together, these findings suggest that symptom reassessment merits prospective evaluation as complementary clinical information, while the hypothetical MRI-omission scenario illustrates possible implications for patients’ out-of-pocket payments.

The clinical rationale is consistent with approaches that emphasize symptom timing and evolution rather than dizziness quality alone [16,17]. HINTS and STANDING have been evaluated for distinguishing central from peripheral causes [23,24,25], but they differ in their intended populations and require appropriate examination and interpretation. Previous studies associated transient symptoms with lower odds of acute brain lesions, and persistent symptoms or absent modulation by head movement with central vertigo [14,26]. In contrast, our study assessed symptom status at routine reassessment, including patients whose subjective symptoms had completely resolved.

Comparison of lesion frequencies across studies requires attention to selection and outcome definitions. Reports from broader ED populations, MRI-selected cohorts, and Japanese patients undergoing CT or MRI have involved different case mixes and definitions of central disease [15,27,28,29]. Our exclusions of headache, cochlear symptoms, and overt focal neurological deficits, together with restriction to MRI recipients, prevent direct generalization to all ED patients with dizziness.

Reassessment requires no additional equipment and can accompany routine clinical evaluation. However, disappearance of symptoms during rest does not imply absence of symptoms during positional changes or walking. BPPV accounted for 88.9% of diagnoses in the complete-resolution group, and conditions that improve when provoking movements are avoided could partly explain the association [17]. Symptom resolution alone cannot diagnose BPPV or another specific peripheral disorder. Recommendations against imaging in patients meeting BPPV criteria depend on a disease-specific assessment, not on symptom disappearance alone [30]. Transient dizziness can precede posterior circulation stroke, and ischemic disease has been identified after transient vestibular symptoms have resolved [31,32]. Imaging and specialist assessment therefore remain important when the history or examination raises concern for a central cause [3,33].

The economic analysis adds the patient perspective to the reported expenditure burden of dizziness imaging [6,7,10]. Individual billing records and copayment rates allowed medical expenditures and patient payments to be evaluated separately, including patients with zero out-of-pocket payments [12,13]. The median estimated payment reduction of JPY 3552 among MRI-omission candidates quantifies a possible initial financial effect, but not its importance to an individual household. Affordability and financial hardship were not assessed. The monetary estimates depend on Japanese tariffs and the selected cohort; they are not directly transferable to other healthcare systems. The approach of evaluating patient payments separately can, however, be adapted using local utilization, prices, and benefit arrangements.

The sensitivity analysis characterized how hypothetical additional outpatient revisit costs could offset the initial reductions. Estimated reductions remained positive across the principal scenarios with additional revisit proportions up to 20%, while the stress scenarios identified break-even thresholds under the four tariff conditions.

Several limitations affect interpretation. This was a retrospective, single-center study restricted to selected MRI recipients. Among 54 patients without MRI, patient preference was recorded for 46, but individual motivations were not established. The lower admission proportion among nonrecipients (20.4% versus 47.6%; Table S2B) demonstrates a difference in cohort composition without explaining MRI decisions. Descriptive comparisons cannot exclude selection by unmeasured factors or determine the direction and magnitude of bias. Institutional recommendations for observation admission also shaped the discharged-home cost-analysis cohort and may limit applicability to other settings.

The outcome was confined to causative lesions detected on initial MRI. Although image interpretation was blinded to post-rest symptom status, attribution of an MRI-detected lesion to the presenting dizziness relied on the final diagnosing physician’s judgment informed by the symptom course, introducing potential outcome-classification bias. Early or small ischemic lesions may be missed [23,34], the symptom-onset-to-imaging interval varied, and repeat MRI was not used to determine the outcome. A first reassessment was documented in only 36 of the 54 hypothetical MRI-omission candidates (66.7%). Recorded reassessments were mainly on the following day, rather than standardized 30- or 90-day follow-up. Events diagnosed elsewhere or outside hospital could have been missed. The 4.0% upper confidence limit for 0/90 refers to the proportion of initial-MRI-detected causative central lesions in that selected group, not the true miss rate of an MRI-omission strategy. Consequently, an acceptable risk of missed disease cannot be established from these observations.

Symptom classification depended on subjective reports and routine documentation, without a standardized severity scale or evaluation of interobserver agreement. Only 12 central lesions occurred, and no multivariable adjustment was performed; age, vascular risk, diagnostic case mix, and examination findings may therefore confound the observed association. Independent predictive value and incremental value beyond established bedside examinations were not established. There was also no no-rest group or comparison of rest durations, so a specific effect of rest cannot be separated from spontaneous symptom change, and the optimal reassessment interval remains unknown.

The cost model held initial non-MRI care and disposition constant. Itemized MRI claims were not reviewed, and deductions used specified 2022 billing assumptions. Additional outpatient revisit costs were modeled rather than observed, with identical assumed revisit proportions applied across candidates. The analysis did not include prolonged observation, alternative tests, inpatient care, treatment after delayed diagnosis, or health consequences of missed disease. Therefore, the present model cannot determine whether the initial estimated reductions would persist if MRI omission resulted in delayed diagnosis or additional downstream care. Costs of visits common to both pathways might also differ with prior imaging. Fee-schedule reductions do not necessarily represent equivalent hospital operating-cost reductions. Unrecorded subsidies and reimbursements, patient and family time, and lost income were not incorporated [35]; therefore, the estimates do not represent full-episode net costs or final household payments.

Prospective multicenter studies should standardize symptom reassessment, evaluate its reproducibility and added diagnostic value, and ascertain missed diagnoses and subsequent healthcare use. Including patient-reported financial and time burdens would clarify whether a selectively imaged pathway offers benefits beyond reductions in initial charges.

## 5. Conclusions

Complete symptom resolution at reassessment was associated with a lower observed proportion of causative central lesions on initial MRI and warrants evaluation as information complementary to established clinical assessment. In a hypothetical scenario, more selective MRI use was estimated to reduce initial medical expenditures and patient out-of-pocket payments. These findings raise the possibility that some MRI examinations may be avoidable in selected patients whose dizziness completely resolves after brief reassessment. In this context, health authorities and insurance claims-review bodies in Japan may have a role in developing and evaluating policies to promote appropriate MRI use and reduce medical expenditures and patient out-of-pocket payments. However, these exploratory findings do not establish the safety of MRI omission; prospective validation incorporating clinical outcomes and downstream healthcare use is required before implementing such policies.

## Figures and Tables

**Figure 1:**
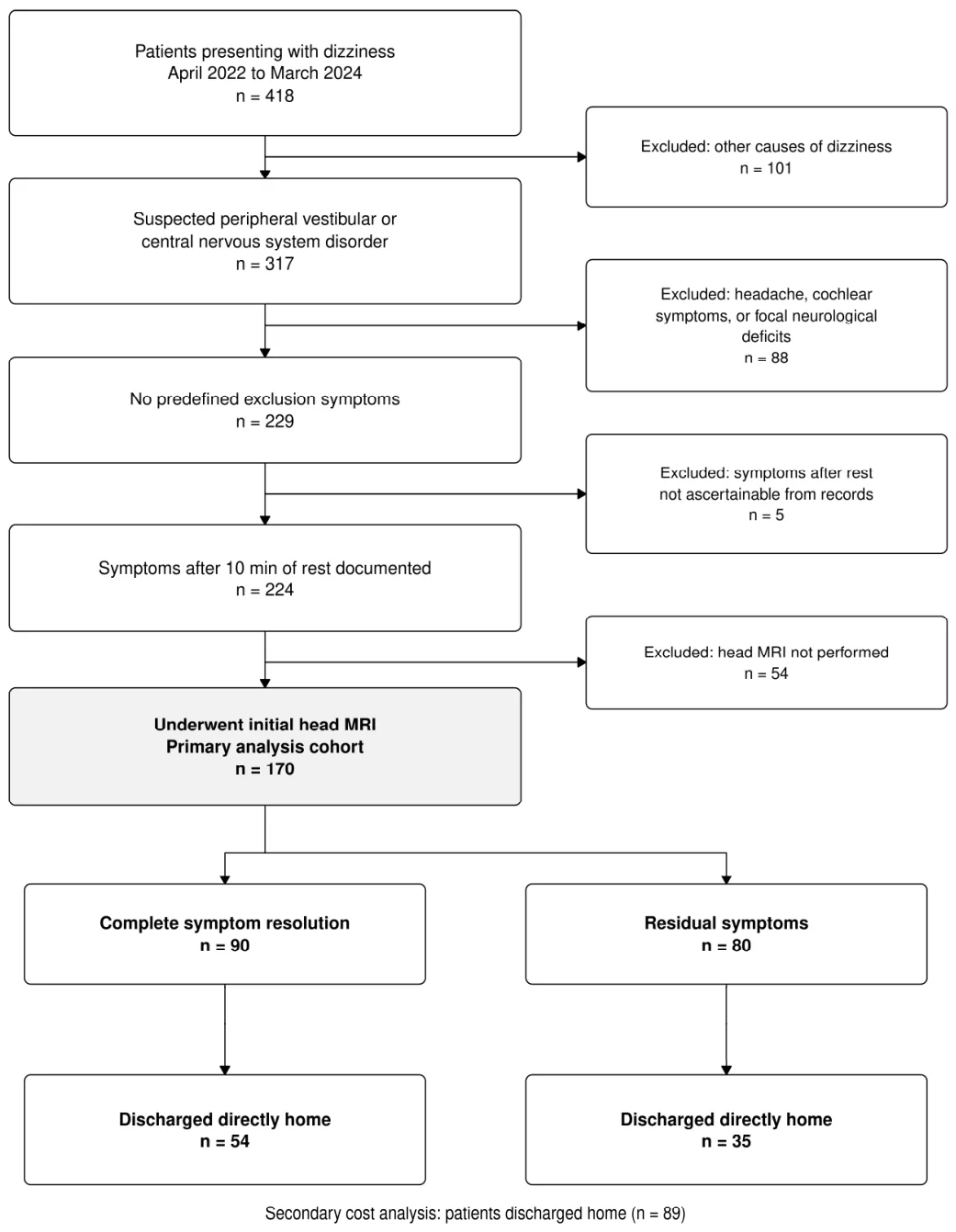
Participant selection and analysis cohorts. Exclusion criteria were applied sequentially in the order shown. Patients who underwent initial head MRI constituted the primary analysis cohort and were classified according to symptom status after 10 min of rest as having complete symptom resolution or residual symptoms. Patients discharged directly home constituted the secondary cohort for the cost analysis. MRI, magnetic resonance imaging.

**Figure 2:**
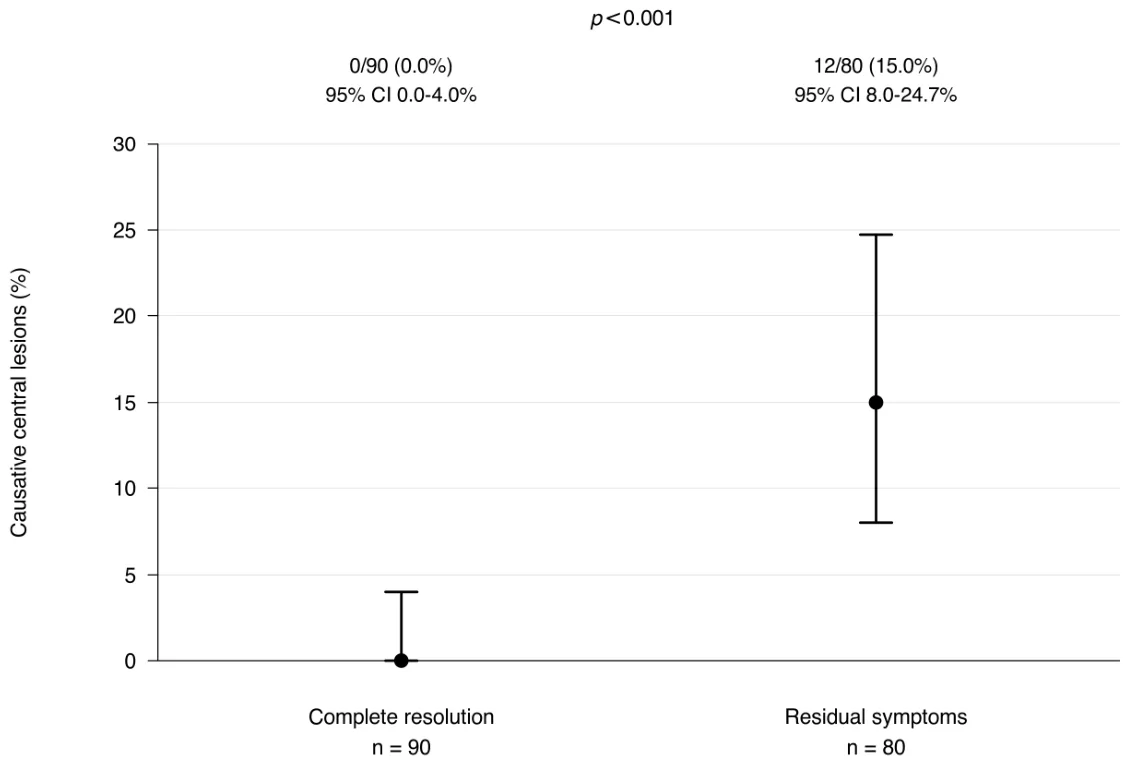
Causative central lesions on initial head MRI according to symptom status after 10 min of rest. Only patients who underwent initial head MRI were included. Residual symptoms included improved but unresolved symptoms and persistent symptoms. Points indicate observed proportions, and vertical bars represent exact 95% confidence intervals calculated using the Clopper–Pearson method. Groups were compared using a two-sided Fisher’s exact test; the *p* value is shown in the figure. MRI, magnetic resonance imaging.

**Table 1:** Patient characteristics and clinical information.

Characteristic	Overall *n* = 170	Complete Resolution *n* = 90	Residual Symptoms *n* = 80
Age, years	71 [57–80]	73 [60–80.8]	65.5 [55.8–79.3]
Male sex	51/170 (30.0)	25/90 (27.8)	26/80 (32.5)
Systolic blood pressure, mmHg	150 [127.3–166]	152 [129–167]	145 [123.8–162.5]
Heart rate, beats/min	75 [68–84]	75.5 [68.3–83.5]	75 [65–84]
Temperature, °C	36.2 [35.8–36.5]	36.2 [35.8–36.4]	36.3 [35.9–36.5]
Respiratory rate, breaths/min	18 [18–20]	18 [18–20]	18 [16–20]
SpO_2_, %	98 [97–98]	98 [97–98]	98 [97–98]
Hospital admission	81/170 (47.6)	36/90 (40.0)	45/80 (56.3)
Discharged directly home	89/170 (52.4)	54/90 (60.0)	35/80 (43.8)
**Copayment rate among patients discharged home**	***n*** ** = 89**	***n*** ** = 54**	***n*** ** = 35**
0%	5/89 (5.6)	2/54 (3.7)	3/35 (8.6)
10%	25/89 (28.1)	19/54 (35.2)	6/35 (17.1)
20%	17/89 (19.1)	12/54 (22.2)	5/35 (14.3)
30%	42/89 (47.2)	21/54 (38.9)	21/35 (60.0)

Continuous variables are presented as median [Q1–Q3], and categorical variables as n/N (%). Copayment rates are summarized only among patients discharged directly from the emergency department; the denominators are shown in the section heading. Symptom groups were defined according to symptom status after 10 min of rest. Between-group hypothesis tests were not performed. There were no missing values within the applicable populations. SpO_2_, peripheral oxygen saturation.

**Table 2:** MRI use and costs among patients discharged home.

Measure	Medical Expenditure	Patient Out-of-Pocket Payment
Actual care, total (*n* = 89)	4,016,030	851,062
Hypothetical scenario, total (*n* = 89)	3,106,730	676,630
Estimated reduction, total	909,300	174,432
Reduction, %	22.6	20.5
Reduction per patient for whom MRI would be omitted, median [Q1–Q3] (*n* = 54)	11,840 [11,840–23,500]	3552 [2240–4248]
Actual care per patient, median [Q1–Q3] (*n* = 89)	44,660 [38,020–51,050]	8930 [5780–13,640]
Hypothetical scenario per patient, median [Q1–Q3] (*n* = 89)	36,720 [22,850–45,200]	6562 [3290–11,228]

MRI examinations: 89 under actual care, 35 under the hypothetical scenario, and 54 omitted (60.7% reduction). All monetary values are in JPY. Values in brackets are Q1–Q3. Total and per-patient cost summaries included all 89 patients who were discharged home. Per-patient reductions among patients for whom MRI would be omitted included 54 patients. Only the initial MRI examination in the complete-resolution group would be omitted under the hypothetical scenario. All other care and discharge decisions were held constant. Medical expenditure covers initial emergency care and is valued at JPY 10 per billing point. Patient out-of-pocket payments exclude hospital-designated out-of-pocket surcharge for after-hours emergency care (sentei ryoyohi). Payments under the hypothetical scenario were rounded to the nearest yen and constrained to a minimum of JPY 0. Reductions were calculated based on individual paired differences. Table S4 lists the billing assumptions. Reduction percentages were calculated using the actual-care total as the denominator. No hypothesis tests or confidence intervals were calculated.

**Table 3:** Two-factor scenario analysis of remaining modeled cost reductions.

**A. Medical Expenditure (JPY)**				
**Assumed Added Revisit Proportion**	**Weekday Daytime**	**After Hours (Special)**	**Holiday**	**Late Night**
0%	909,300	909,300	909,300	909,300
1%	899,114	896,468	896,414	895,172
2%	888,928	883,636	883,528	881,044
5%	858,369	845,139	844,869	838,659
10%	807,438	780,978	780,438	768,018
20%	705,576	652,656	651,576	626,736
30%	603,714	524,334	522,714	485,454
40%	501,852	396,012	393,852	344,172
50%	399,990	267,690	264,990	202,890
60%	298,128	139,368	136,128	61,608
70%	196,266	11,046	7266	−79,674
80%	94,404	−117,276	−121,596	−220,956
90%	−7458	−245,598	−250,458	−362,238
100%	−109,320	−373,920	−379,320	−503,520
**B. Patient out-of-pocket payments (JPY)**				
**Assumed Added Revisit Proportion**	**Weekday Daytime**	**After Hours (Special)**	**Holiday**	**Late Night**
0%	174,432	174,432	174,432	174,432
1%	172,476	171,957	171,946	171,702
2%	170,520	169,481	169,460	168,972
5%	164,652	162,055	162,002	160,783
10%	154,871	149,677	149,571	147,133
20%	135,310	124,922	124,710	119,834
30%	115,749	100,167	99,849	92,535
40%	96,188	75,412	74,988	65,236
50%	76,627	50,657	50,127	37,937
60%	57,066	25,902	25,266	10,638
70%	37,505	1147	405	−16,661
80%	17,944	−23,608	−24,456	−43,960
90%	−1617	−48,363	−49,317	−71,259
100%	−21,178	−73,118	−74,178	−98,558

Values are the modeled initial-visit expenditure or patient-payment reductions remaining after expected additional outpatient revisit costs: initial reduction minus the assumed additional revisit proportion multiplied by the patient-level modeled revisit costs. Negative values indicate a modeled increase in costs. Patient payments reflect individual recorded copayment rates, rather than a single population-average rate. Assumed additional revisit proportions of 0%, 1%, 2%, 5%, 10%, and 20% are the baseline and principal deterministic scenarios; 30–100% (in 10-percentage-point increments) are stress-test assumptions used to identify break-even conditions, not predicted revisit rates. These inputs were author-specified and were not estimated from observed follow-up or external studies. The model includes only an additional outpatient visit with blood tests and MRI, as detailed in Table S5. Admission, delayed diagnosis, missed-event harms, and other downstream or indirect costs were not modeled. Healthcare visits that would have occurred regardless of initial MRI use were not modeled. This conditional analysis does not establish MRI-omission safety or net savings over the full episode of care. ED, emergency department; JPY, Japanese yen; MRI, magnetic resonance imaging.

## Data Availability

The datasets generated and/or analyzed during the current study are not publicly available because they contain potentially identifiable patient information and are subject to institutional and ethical restrictions. Data may be available from the corresponding author upon reasonable request and with appropriate institutional and ethical approval.

## Supplementary Materials

The following supporting information can be downloaded at: https://www.mdpi.com/article/10.3390/healthcare14213784/s1, Table S1A: Final diagnoses by symptom group; Table S1B: Departments by symptom group; Table S2A: Reported reasons for not undergoing MRI; Table S2B: Characteristics by initial head MRI status; Table S3: First reassessment after negative initial MRI; Table S4: MRI tariff assumptions for patients discharged home; Table S5: Modeled additional outpatient revisit costs and break-even thresholds.

## Abbreviations

The following abbreviations are used in this manuscript:

AI	Artificial intelligence
BPPV	Benign paroxysmal positional vertigo
CHEERS	Consolidated Health Economic Evaluation Reporting Standards
CI	Confidence interval
CT	Computed tomography
ED	Emergency department
GRACE-3	Guidelines for Reasonable and Appropriate Care in the Emergency Department 3
HINTS	Head impulse, nystagmus, and test of skew
JPY	Japanese yen
MRI	Magnetic resonance imaging
SpO_2_	Peripheral oxygen saturation
STROBE	Strengthening the Reporting of Observational Studies in Epidemiology
STANDING	Bedside algorithm assessing spontaneous/positional nystagmus, nystagmus direction, head impulse, and standing/gait
TiTrATE	Timing, triggers, and targeted examinations
USD	United States dollar
