# Supplementary material for: Complete Symptom Resolution After Rest in Acute Dizziness: Implications for Selective Magnetic Resonance Imaging Use and Patient Out-of-Pocket Costs in Japan
Source: Healthcare (Basel). 2026 Nov 5;14(21):3784. doi: 10.3390/healthcare14213784 (PMC13763052; doi:10.3390/healthcare14213784)
Supplement: Supplementary file 1 [file healthcare-14-03784-s001.zip › healthcare-4627457-supplementary.pdf]

# Supplementary Materials

## Complete Symptom Resolution After Rest in Acute Dizziness: Implications for Selective Magnetic Resonance Imaging Use and Patient Out-of-Pocket Costs in Japan

Kazuki Kikuchi et al.

### Table S1A. Final diagnoses by symptom group

| Final diagnosis | Overall<br>n = 170 | Complete resolution<br>n = 90 | Residual symptoms<br>n = 80 |
|---|---|---|---|
| **Noncentral diagnoses** | **158/170 (92.9)** | **90/90 (100.0)** | **68/80 (85.0)** |
| Benign paroxysmal positional vertigo (BPPV) | 106/170 (62.4) | 80/90 (88.9) | 26/80 (32.5) |
| Vestibular neuritis | 17/170 (10.0) | 0/90 (0.0) | 17/80 (21.3) |
| Ménière disease | 6/170 (3.5) | 1/90 (1.1) | 5/80 (6.3) |
| Peripheral dizziness of unknown origin | 29/170 (17.1) | 9/90 (10.0) | 20/80 (25.0) |
| **Central nervous system disorders** | **12/170 (7.1)** | **0/90 (0.0)** | **12/80 (15.0)** |
| Cerebral infarction | 6/170 (3.5) | 0/90 (0.0) | 6/80 (7.5) |
| Brain tumor | 3/170 (1.8) | 0/90 (0.0) | 3/80 (3.8) |
| Vertebral artery dissection | 1/170 (0.6) | 0/90 (0.0) | 1/80 (1.3) |
| Cerebral hemorrhage | 1/170 (0.6) | 0/90 (0.0) | 1/80 (1.3) |
| Basilar artery stenosis | 1/170 (0.6) | 0/90 (0.0) | 1/80 (1.3) |

Values are presented as n/N (%). Percentages for subtotals and individual diagnoses use all patients in the corresponding symptom group as the denominator.

Diagnostic categories are summarized according to the recorded central/noncentral classification, with subtotals followed by their component diagnoses.

Complete symptom resolution and residual symptoms refer to symptom status after 10 min of rest.

There were no missing final diagnoses or central/noncentral classifications. Between-group hypothesis tests were not performed.

BPPV, benign paroxysmal positional vertigo.

## Table S1B. Departments by symptom group

| Department/service | Overall | Complete symptom resolution | Residual symptoms |
|---|---|---|---|
| **Department making the final diagnosis** | **n = 170** | **n = 90** | **n = 80** |
| Otorhinolaryngology | 122 (71.8) | 71 (78.9) | 51 (63.8) |
| Emergency medicine | 36 (21.2) | 19 (21.1) | 17 (21.3) |
| Neurology | 8 (4.7) | 0 (0.0) | 8 (10.0) |
| Neurosurgery | 4 (2.4) | 0 (0.0) | 4 (5.0) |
| **Admitting service** | **n = 81** | **n = 36** | **n = 45** |
| Otorhinolaryngology | 67 (82.7) | 35 (97.2) | 32 (71.1) |
| Neurology | 9 (11.1) | 0 (0.0) | 9 (20.0) |
| Neurosurgery | 2 (2.5) | 0 (0.0) | 2 (4.4) |
| General internal medicine | 3 (3.7) | 1 (2.8) | 2 (4.4) |

Values are presented as n (%). The section headings indicate the applicable denominators for each column.

Diagnostic departments included all patients, whereas admitting services included only admitted patients.

General internal medicine does not include neurology.

The department making the final diagnosis and the admitting service were recorded separately and may have differed.

There were no missing values in the applicable populations. Between-group hypothesis tests were not performed.

## Table S2A. Reported reasons for not undergoing MRI

| Reported reason | n | % |
|---|---|---|
| Inability to remove a ring | 1 | 1.9 |
| Tattoo | 1 | 1.9 |
| Implanted metal or device (including pacemaker) | 3 | 5.6 |
| Claustrophobia | 3 | 5.6 |
| Patient preference | 46 | 85.2 |

Percentages use all 54 patients without an initial head MRI as the denominator.

The specific reasons underlying patient preferences were not available.

Percentages may not sum to 100% because of rounding.

MRI, magnetic resonance imaging.

## Table S2B. Characteristics by initial head MRI status

| Characteristic | Initial MRI performed<br>n = 170 | Initial MRI not performed<br>n = 54 |
|---|---|---|
| Age, years | 71 [57–80] | 69 [54–78] |
| Male sex | 51/170 (30.0) | 16/54 (29.6) |
| Systolic blood pressure, mmHg | 150 [127.3–166] | 141.5 [121.8–156.5] |
| Heart rate, beats/min | 75 [68–84] | 81 [70.3–87.5] |
| Temperature, °C | 36.2 [35.8–36.5] | 36.2 [36.0–36.4] |
| Respiratory rate, breaths/min | 18 [18–20] | 18 [18–20] |
| $SpO_2$, % | 98 [97–98] | 98 [97–98] |
| Complete symptom resolution | 90/170 (52.9) | 32/54 (59.3) |
| Residual symptoms | 80/170 (47.1) | 22/54 (40.7) |
| Hospital admission | 81/170 (47.6) | 11/54 (20.4) |
| **Department making the final diagnosis** | | |
| Otorhinolaryngology | 122/170 (71.8) | 32/54 (59.3) |
| Emergency medicine | 36/170 (21.2) | 22/54 (40.7) |
| Neurology | 8/170 (4.7) | 0/54 (0.0) |
| Neurosurgery | 4/170 (2.4) | 0/54 (0.0) |

Continuous variables are presented as median [Q1–Q3], and categorical variables as n/N (%).

Both groups met the clinical eligibility criteria for MRI. Patients who did not undergo MRI were excluded from the primary analysis.

Symptom status was assessed after 10 min of rest. Residual symptoms included improved but unresolved symptoms and persistent symptoms.

There were no missing values in the applicable populations for any of the variables.

Between-group hypothesis tests were not performed.

MRI, magnetic resonance imaging; $SpO_2$, peripheral oxygen saturation.

## Table S3. First reassessment after negative initial MRI

### A. By symptom group

| | Overall n = 158 | Complete symptom resolution n = 90 | Residual symptoms n = 68 |
|---|---|---|---|
| Documented first reassessment | 125/158 (79.1) | 72/90 (80.0) | 53/68 (77.9) |
| First reassessment within 7 days | 124/158 (78.5) | 72/90 (80.0) | 52/68 (76.5) |
| First reassessment after 7 days | 1/158 (0.6) | 0/90 (0.0) | 1/68 (1.5) |
| Time to first reassessment, days, median [Q1–Q3] | 1 [1–1] | 1 [1–1] | 1 [1–1] |
| Time to first reassessment, days, range | 1–12 | 1–7 | 1–12 |
| New central nervous system diagnosis at first reassessment | 0/125 (0.0) | 0/72 (0.0) | 0/53 (0.0) |

### B. By disposition

| | Overall n = 158 | Admitted n = 70 | Discharged home n = 88 |
|---|---|---|---|
| Documented first reassessment | 125/158 (79.1) | 70/70 (100.0) | 55/88 (62.5) |
| First reassessment within 7 days | 124/158 (78.5) | 70/70 (100.0) | 54/88 (61.4) |
| First reassessment after 7 days | 1/158 (0.6) | 0/70 (0.0) | 1/88 (1.1) |
| Time to first reassessment, days, median [Q1–Q3] | 1 [1–1] | 1 [1–1] | 1 [1–3] |
| Time to first reassessment, days, range | 1–12 | 1–1 | 1–12 |
| New central nervous system diagnosis at first reassessment | 0/125 (0.0) | 0/70 (0.0) | 0/55 (0.0) |

Only patients without a causative central lesion on the initial MRI were included. Values are n/N (%), median [Q1–Q3], or range.
Reassessment included inpatient review and outpatient revisits by a relevant department; the day after the emergency visit was defined as day 1. Timing summaries included only patients with a documented first reassessment.
For the new central nervous system diagnosis, denominators were the patients with a documented first reassessment (125 overall; 72 and 53 by symptom group; 70 and 55 by disposition).
Reassessment was not documented for 33 patients: 18 with complete symptom resolution and 15 with residual symptoms; all 33 patients were discharged. Patient outcomes were not imputed.
MRI, magnetic resonance imaging.

## Table S4. MRI tariff assumptions for patients discharged home

| Component | Preceding head CT | No CT; no emergency add-on | No CT; emergency add-on |
|---|---|---|---|
| Patients whose MRI is omitted, n | 30 | 9 | 15 |
| MRI acquisition, points | 1,064 | 1,330 | 1,330 |
| Electronic image management, points | 120 | 120 | 120 |
| Diagnostic fee, points | 0 | 450 | 450 |
| Image management add-on, points | 0 | 340 | 340 |
| Emergency imaging add-on, points | 0 | 0 | 110 |
| **Total deduction, points** | **1,184** | **2,240** | **2,350** |
| **Medical expenditure reduction per patient, JPY** | **11,840** | **22,400** | **23,500** |
| **Medical expenditure reduction for stratum, JPY** | **355,200** | **201,600** | **352,500** |

Only the 54 patients whose initial MRI examinations were hypothetically omitted are shown. These are model assumptions and do not verify the itemized billing components.

Under the 2022 fee schedule, 1 point was equivalent to JPY 10 for MRI performed using a 1.5-T scanner. For patients with a preceding head CT, MRI acquisition was valued at 80% of the standard 1,330 points (1,330 x 0.8 = 1,064 points); CT-related diagnostic and management fees were retained.

The 110-point emergency imaging add-on was deducted only when recorded; no CT was performed in these cases, and omission of MRI removed the add-on.

Scenario patient payment = max(0, round to nearest yen [actual payment excluding selected medical care fees - MRI deduction × recorded co-payment rate]). Half-yen values were rounded upward.

Patient-level reduction was calculated as actual payment minus scenario payment. Unrecorded reimbursement adjustments were not modeled. No reduction in inpatient costs was modeled because the cost analysis was restricted to patients discharged home.

CT, computed tomography; MRI, magnetic resonance imaging.

## Table S5. Modeled additional outpatient revisit costs and break-even thresholds

### A. Fee components by revisit timing and initial head CT status

| Component (points unless specified) | Weekday daytime | After hours (special) | Holiday | Late night |
|---|---|---|---|---|
| Outpatient visit fee (A002) | 74 | 74 | 74 | 74 |
| Time-related consultation add-on | 0 | 180 | 190 | 420 |
| Blood-test panel | 159 | 159 | 159 | 159 |
| Emergency laboratory add-on | 0 | 200 | 200 | 200 |
| Emergency imaging add-on | 0 | 110 | 110 | 110 |
| MRI-related fees: preceding CT (n = 30) | 1,184 | 1,184 | 1,184 | 1,184 |
| **Total points: preceding CT** | **1,417** | **1,907** | **1,917** | **2,147** |
| **Added revisit cost, JPY: preceding CT** | **14,170** | **19,070** | **19,170** | **21,470** |
| MRI-related fees: no preceding CT (n = 24) | 2,240 | 2,240 | 2,240 | 2,240 |
| **Total points: no preceding CT** | **2,473** | **2,963** | **2,973** | **3,203** |
| **Added revisit cost, JPY: no preceding CT** | **24,730** | **29,630** | **29,730** | **32,030** |

### B. Patient-weighted costs and break-even additional revisit proportions

| Revisit condition | Medical cost per revisit, JPY | Patient payment per revisit, JPY | Medical break-even, % | Patient break-even, % |
|---|---|---|---|---|
| Weekday daytime | 18,863 | 3,622 | 89.27 | 89.17 |
| After hours (special) | 23,763 | 4,584 | 70.86 | 70.46 |
| Holiday | 23,863 | 4,604 | 70.56 | 70.16 |
| Late night | 26,163 | 5,055 | 64.36 | 63.90 |

Revisit costs are modeled for the 54 discharged patients with complete symptom resolution whose initial MRI was hypothetically omitted. Each scenario assumes an additional outpatient ED visit at the same institution on a separate day of the same calendar month, with a fixed blood-test panel and noncontrast 1.5-T head MRI. No subsequent admission is modeled.

The blood-test panel comprises venous sampling (37 points), biochemistry (10 or more items; 106 points), and C-reactive protein (16 points); complete blood count is considered included in the outpatient visit fee (0 additional points). Specimen test interpretation fees were assumed already billed during the initial visit and were not billed again.

MRI-related points follow the 2022 Japanese fee schedule and Table S4: 1,184 points after initial CT (MRI acquisition at 80%, plus electronic image management), and 2,240 points without initial CT (including diagnostic and management fees). Time-related and urgent laboratory/imaging add-ons were assumed to meet the applicable billing conditions; add-ons were not duplicated. The after-hours scenario used the 180-point special after-hours consultation add-on.

Patient-weighted costs reflect 30 patients with and 24 without initial CT, combined with individual recorded copayment rates. The initial modeled reductions were JPY 909,300 (medical expenditure) and JPY 174,432 (patient payments), giving mean break-even added costs of JPY 16,838.89 and JPY 3,230.22 per MRI-omission candidate, respectively. Displayed costs are rounded; break-even proportions were calculated from unrounded totals.

CT, computed tomography; ED, emergency department; JPY, Japanese yen; MRI, magnetic resonance imaging.
